# Biomarker-driven management strategies for peripheral T cell lymphoma

**DOI:** 10.1186/s13045-020-00889-z

**Published:** 2020-05-24

**Authors:** Erin Mulvey, Jia Ruan

**Affiliations:** Meyer Cancer Center, Weill Cornell Medicine, 1305 York Avenue, 7th Floor, New York, NY 10021 USA

**Keywords:** CD30-positive peripheral T cell lymphoma (PTCL), Nodal PTCL with T follicular helper (TFH) phenotype, Epigenetic targeting, HDAC inhibitors, Hypomethylating agents, Tumor microenvironment

## Abstract

Peripheral T cell lymphomas are heterogeneous diseases which remain treatment challenges. Recent advances in molecular and genomic profiling have provided unprecedented insight into disease pathogenesis driven by distinct cells of origins and molecular pathways. The discovery and clinical application of molecular biomarkers in PTCL subtypes has the potential to transform personalized care for patients with PTCL in diagnosis, prognosis, and therapy. Targeting CD30+ PTCL with the antibody-drug conjugate brentuximab vedotin in the relapsed setting and in combination with chemotherapy in the frontline setting has improved patient survivals. Epigenetic modifying agents, including HDAC inhibitors and hypomethylating agents, have demonstrated broad clinical efficacy and durability and are in clinical development for combination strategies for both relapsed and frontline settings. Wide-ranging novel agents targeting critical intracellular pathways and tumor microenvironment are in active exploration to define clinical activities. This review summarizes PTCL-specific biomarkers which are increasingly incorporated in clinical practice to guide precision diagnosis and personalized treatment.

## Introduction

Peripheral T cell lymphomas (PTCL) are a heterogeneous group of non-Hodgkin lymphomas (NHL) derived from mature T/NK cells, encompassing 5–10% NHL in Western countries with a higher incidence of 15–20% in Asia and South America [1, 2]. Despite divergent cells of origin and mechanisms of lymphomagenesis, management of PTCL has historically followed the treatment framework for aggressive B cell NHL, in part due to lack of understanding of subtype-specific disease pathogenesis, as well as difficulty of conducting dedicated prospective treatment studies. Although generally not curative with the exception of ALK+ ALCL, cyclophosphamide, doxorubicin, vincristine, prednisone (CHOP) is the most commonly prescribed initial treatment for systemic PTCL. While autologous stem cell transplant may extend progression-free survival (PFS) for some patients, relapse remains common. Recent advances in the molecular and genetic bases of PTCL biology, as well as emergence of targeted therapeutic agents, have made it possible to envision personalized therapeutic progress.

## Biomarker-driven classification of PTCL

There are over 30 different subtypes of peripheral T cell lymphoma in the 2016 WHO classification of lymphoid malignancies. The most prevalent nodal T cell lymphomas are peripheral T cell lymphoma, not otherwise specified (PTCL-NOS); nodal T cell lymphoma with T follicular helper (TFH) phenotype which includes angioimmunoblastic T cell lymphoma (AITL); and systemic anaplastic large cell lymphoma (sALCL). Less common subtypes include extranodal NK/T cell lymphoma, nasal type (ENKTL), adult T cell lymphoma/leukemia (ATLL), enteropathy-associated T cell lymphoma (EATL), hepatosplenic T cell lymphoma (HSTL), and subcutaneous panniculitis-like T cell lymphoma (SPTL), amongst others. The complexity of PTCL reflects divergent cells of origin and mechanisms of lymphomagenesis.

### PTCL, not otherwise specified (NOS)

PTCL, NOS is the most common subtype of PTCL, accounting for approximately 30% of PTCL in Western countries and approximately 20–25% in Asia [1]. There is no characteristic immunophenotype for PTCL, NOS. T cell-associated antigens such as CD2, CD3, CD5, and CD7 are variably expressed; one or more antigens such as CD5 or CD7 are frequently lost. Tumor cells can express either CD4 or CD8, with the majority expressing CD4. Clonal T cell receptor (TCR) gene rearrangements are usually detected and mostly show alpha/beta T cell receptors (TCR beta positive). Gene expression profiles (GEPs) have identified two subgroups of PTCL, NOS, with distinct gene expression driven by the transcription factors TBX-21 or GATA-3, which promote differentiation of CD4+ T cells into TH1 and TH2 helper cells, respectively. The GATA3 subgroup was significantly associated with poor survival outcome [3, 4].

### Systemic anaplastic large cell lymphoma

Systemic anaplastic large cell lymphoma (sALCL) is the second most common PTCL subtype and accounts for 24% of PTCL in the USA, with less incidence in Europe and Asia [2, 5]. There are three distinct forms of sALCL, including ALK-positive ALCL which has a peak incidence in children and young adults and is associated with translocations involving ALK gene located on chromosome 2p23, ALK-negative ALCL that typically presents in older adults, and the breast implant-associated ALK-negative sALCL. The immunophenotype of ALCL is notable for universal expression of CD30, frequent expression of TIA1, granzyme B, perforin, EMA, and low expression of CD8 and CD56. The majority of ALCL have clonally rearranged TCR genes, while roughly 10% in the null group have no rearrangement of TCR. Cytogenetic studies play a key role in ALCL diagnosis. ALK-positive ALCL most frequently has t(2;5) which fuses nucleophosmin (NPM) gene on chromosome 5q35 with the *ALK* gene on chromosome 2p23. NPM-ALK is an oncogenic tyrosine kinase which promotes signaling of JAK/STAT pathway. Less frequent variant *ALK* rearrangements include t(1;2) and t(2;3). In ALK-negative ALCL, recurrent chromosomal rearrangements involving the DUSP22-IRF4 locus on 6p25.3 were associated with favorable outcomes, while those involving TP53 homolog TP63 on 3q28 were associated with aggressive clinical behavior and poor outcomes [6]. Gene expression signatures of ALCL showed hyper-activation of STAT3 due to rearrangements of ALK tyrosine kinase or activating mutations in the JAK/STAT pathway.

### Nodal PTCL with T follicular helper phenotype

The 2016 WHO revision brings together T cell lymphoma subtypes including angioimmunoblastic T cell lymphoma, follicular T cell lymphoma (FTCL), and PTCL with T follicular phenotype under the provisional entity of nodal PTCL with TFH phenotype, which shared TFH-related antigens and recurrent genetic abnormalities. AITL is one of the more common PTCLs encountered in Western countries, accounting for ~ 28% PTCL in Europe, with lower incidence in North America and Asia (~ 15%) [7]. Patients typically present with advanced-stage disease and symptoms of a systemic illness such as rash, fever, and malaise. AITL can also manifest with immunologic abnormalities such as polyclonal hypergammaglobulinemia or autoimmune cytopenias. The histology of AITL is characterized by a polymorphous infiltrate of immune cells with a prominent proliferation of high endothelial venules. The tumor cells express follicular T helper cell markers including CD10, CXCL13, PD-1, BCL6, and ICOS. Molecular studies show that T cell receptor genes are rearranged in 75 to 90% of cases, while immunoglobulin heavy chains may be rearranged in up to 25% due to expansion of Epstein-Barr virus (EBV)-associated immunoblastic B cell clones. Gene expression profiling demonstrates a molecular signature typical of follicular helper T cell origin [8, 9], with recurrent driver mutations in *TET2*, *IDH2*, *DNMT3A*, and *RHOA*, which are implicated in epigenetic regulation.

### Extranodal natural killer/T cell lymphoma, nasal type

ENKTL is most common in Asia and South America, where EBV infection is endemic. Virtually, all cases contain EBV-encoded small nuclear RNAs (EBERS) [2, 10]. Clinical presentation is notable for mid-facial extra-nodal lymphoma associated with vascular invasion and necrosis. The immunophenotype of ENKTL is similar to that of natural killer cells which express CD2, CD56, and cytotoxic granule proteins such as granzyme B, TIA-1, and perforin, and lack surface T cell receptor (TCR). There is no typical diagnostic cytogenetic change in ENKTL.

### Adult T cell leukemia-lymphoma

ATLL is a peripheral T cell lymphoma subtype associated with infection by human T cell lymphotropic virus type 1 (HTLV-1) of CD4 cells in endemic areas such as southern Japan and the Caribbean basin [11]. The long-term risk of developing ATLL following HTLV-I infection has been estimated to be 4 to 5%, usually after a latency period of several decades. Several clinical variants of ATLL have been described, including acute, lymphomatous, chronic, and smoldering. Peripheral blood smears from leukemia patients exhibit bizarre hyperlobated nuclei known as “clover leaf” or “flower cells.” For immunophenotype, the origin of the malignant cell in ATLL is an HTLV-I-infected memory CD4+ T lymphocyte which expresses CD2, CD3, CD4, and CD25 in most cases. CD52 is often expressed. Genetic sequencing showed somatic mutations in *RHOA* and *TET2*, loss-of-function mutations in *TP53*, and overexpression of *PD-L1* [12].

## Biomarker-driven therapeutic strategies in R/R PTCL

**Fig. 1:**
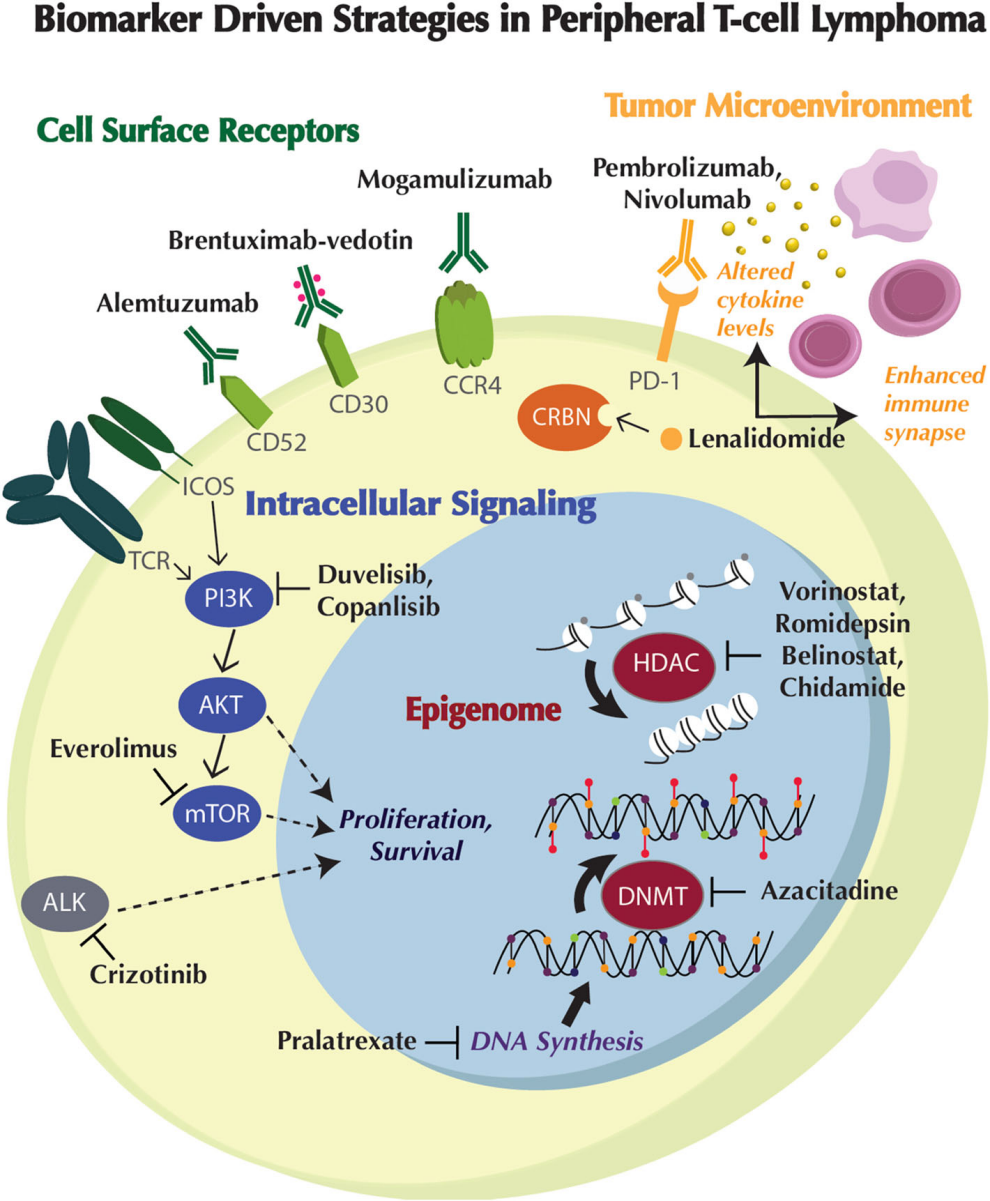
Biomarker-driven strategies in peripheral T cell lymphoma. Positive and inhibitory interactions are depicted as solid arrows and bar-headed lines, respectively. The protein symbols of genes appear inside colored ovals. ALK, oncogenically activated anaplastic lymphoma kinase. AKT, protein kinase B. CCR4, chemokine receptor 4. CD30, cluster of differentiation 30. CD52, cluster of differentiation 52. CRBN, cereblon. DNMT, DNA methyltransferase. HDAC, histone deacetylase. ICOS, inducible T cell co-stimulator. mTOR, mammalian target of rapamycin. PD-1, programmed death receptor 1. PI3K, phophoinositide 3-kinase. TCR, T cell receptor

In addition to contribution to classification and diagnosis of PTCL subtypes, biomarkers provide critical insights into the pathogenic pathways and biological rationale for novel therapeutic intervention (Fig. 1, Tables 1, 2, 3, and 4).

### Targeting cell surface receptors

#### CD30

CD30 is a tumor necrosis factor (TNF) receptor family transmembrane receptor with restricted expression in activated T and B cells in normal lymphoid tissues [13]. Across PTCL subtypes, systemic ALCLs universally express CD30 in membrane and golgi patterns, while approximately 50% of non-ALCL subtypes, including PTCL, NOS, AITL, ENKTL, ATLL, EATL, and HSTL, express CD30 at variable levels. CD30 expression in PTCL suggests a CD30-mediated pathogenic mechanism for potential therapeutic targeting with brentuximab vedotin (BV), an antibody-drug conjugate that combines cytotoxic monomethylauristatin E (MMAE) with an anti-CD30 antibody [14]. The level of CD30 expression may not always be predictive of response to BV due in part to tumor heterogeneity, variation in detection and reporting of CD30 expression, and proposed secondary mechanisms of action. Single-agent BV given at 1.8 mg/kg intravenously every 3 weeks for up to 16 cycles in 58 patients with relapsed or refractory (R/R) sALCL showed overall response rate (ORR) of 86% with 57% complete response (CR) in a pivotal phase 2 study, leading to approval in the USA, EU, and Japan for R/R sALCL [15]. Responses in sALCL appear to be durable: 5-year overall survival (OS) and progression-free survival (PFS) were 79% and 57%, respectively, in patients who achieved a CR. In patients who did not achieve a CR, the 5-year OS was 25% [15]. Grade 3 or 4 adverse events (AEs) experienced by ≥ 10% of patients were neutropenia (21%), thrombocytopenia (14%), and peripheral sensory neuropathy (12%). Overall, treatment was well tolerated. In non-ALCL, single-agent BV showed clinical antitumor activity in AITL and PTCL-NOS, with ORR of 41% (including 54% ORR in AITL with a median PFS of 6.7 months), although responses did not correlate with CD30 expression [16]. These results with single-agent BV paved the way for clinical development of BV-containing combinations in both relapsed and frontline settings.

#### CD52

CD52 is a glycosylphosphatidylinositol (GPI) anchored low molecular weight glycoprotein (21–28 kDa) expressed on the surface of B and T lymphocytes, natural killer cells, monocytes, macrophages, and some dendritic cells [17]. PTCL subtypes such as PTCL/NOS, AITL, ATLL, HSTL, and T-PLL were shown to have high frequency of CD52 expression (> 90%) by flow cytometry and IHC analysis, while CD52 expression was low in ALCL and ENT/NKCL [18, 19]. Although the variable and differential CD52 expression in T cell lymphoma tumor cells implies a rational role for anti-CD52 mAb alemtuzumab in the treatment of PTCL, persistent expression of CD52 by the background normal T- and B cell infiltrate limits therapeutic window of anti-CD52 therapy due to potential immuno-suppression, including increased risk of viral and other opportunistic infections [20].

Single-agent alemtuzumab has established treatment efficacy in T cell prolymphocytic leukemia (T-PLL). Phase I/II and retrospective studies have indicated response rates of up to 75% in patients with previously untreated disease, although relapse remains nearly universal with mOS of < 2 years. When given three times weekly at 3, 10, and 30 mg IV during week one, followed by 30 mg three times a week for up to 16 weeks, response rate was over 90% in 41 previously untreated T-PLL patients with 81% achieving CRs [21]. Prophylaxis against pneumocystis jiroveci (PJP) and herpes viruses together with regular monitoring for CMV reactivation are recommended to minimize the risk for serious infections [22].

#### CCR4

CC chemokine receptor 4 (CCR4) promotes memory T cell homing to peripheral tissues and is preferentially expressed by Th2 and regulatory T cell subsets [24]. In addition to expression in most CD4+CD25+ ATLL, CCR4 is expressed in approximately 30–65% of PTCL tumor cells, including high expression (~ 65%) in ALK-negative ALCL, variable expression (30–40%) in PTCL/NOS, AITL, and transformed MF, while rarely expressed in ENKTL or ALK-positive ALCL [25]. Mogamulizumab (KW-0761) is a defucosylated, humanized, IgG1 monoclonal antibody directed against CCR4 that has direct cytotoxic effect on CCR4-positive lymphoma cells via ADCC, as well as immunomodulatory potential by depletion of regulatory T cells to enhance anti-tumor immunity. Mogamulizumab is approved in Japan for R/R CCR4+ ATLL and CTCL and has been approved in the USA for R/R MF and Sezary syndrome. In 28 Japanese patients with CCR4+ R/R ATLL treated with mogamulizumab at 1.0 mg/kg intravenously once weekly for 8 weeks demonstrated ORR of 50% with 30% CR, and median PFS and OS of 5.2 and 13.7 months, respectively [26]. The most common AEs were hematologic events, pyrexia, and skin reactions, all of which were reversible and manageable.

### Targeting the epigenome

Emerging genetic studies with gene expression profiling (GEP) and next-generation sequencing (NGS) have shown recurrent mutations in epigenetic regulators which contribute to lymphomagenesis and may represent the target of tailored therapies such as epigenetic modifiers [2].

#### HDAC inhibitors

In preclinical studies using TCL cell lines, HDAC inhibitors were shown to induce cell-cycle arrest, apoptosis, and DNA damage and modulate a number of intracellular pathways [28]. Vorinostat, a pan HDAC, was the first drug of its class to be approved by the FDA in 2006 for the treatment of cutaneous T cell lymphoma. A single-arm open-label phase II trial enrolled 74 patients with stage IB or greater CTCL who had failed 2 or more lines of systemic therapy including bexarotene. Patients were treated with oral vorinostat at 300 mg daily or 300 mg 5 days a week and demonstrated favorable responses and an acceptable safety profile: ORR 30% and a median time to tumor progression 202 days [29]. This approval ushered in a still ongoing period of research and development of epigenetic therapy, particularly in TCL.

Romidepsin is a bicyclic class 1 selective HDAC inhibitor approved by the FDA for treatment in patients with R/R PTCL who have failed at least one prior systemic therapy. In a pivotal phase 2 study, 130 patients received romidepsin at 14 mg/m^2^ on days 1, 8, and 15 every 28-day cycle, which showed ORR of 25% including 15% CR/unconfirmed CR (CRu), with durable median duration of response (DOR) of 28 months in responders [30, 31]. Adverse events were manageable and consistent with other HDAC inhibitors, with thrombocytopenia (24%), neutropenia (20%), and infections (all types, 19%) representing the most common grade ≥ 3 AEs. Given the promising results of this study, romidepsin has also been combined with other agents, such as lenalidomide [32], carfilzomib [33], or pralatrexate [34], in attempts to improve efficacy. A phase I/II trial, which included 11 patients with R/R PTCL, showed an ORR of 50% to the doublet of romidepsin given at MTD of 14 mg/m^2^ IV on days 1, 8, and 15 and lenalidomide 25 mg orally on days 1–21 of 28-day cycles [32]. Romidepsin has additionally been combined with lenalidomide and carfilzomib as a triplet in a phase Ib/IIa study that included 16 patients with R/R PTCL. MTD was defined as romidepsin 8 mg/m^2^ days 1 and 8, lenalidomide 15 mg days 1–15, and carfilzomib 36 mg/m^2^ days 1 and 8, every 21-day cycle. The triplet demonstrated a 50% ORR with 30% CR, which seemed to be enriched among patients with AITL (4/5 patients) [33]. A phase I trial combining romidepsin 15 mg/m^2^ and pralatrexate 25 mg/m^2^ in 18 patients with R/R PTCL showed ORR of 71%, which has led to an ongoing phase II trial (NCT01947140) in PTCL [34].

Belinostat, a hydroxamic acid-derived pan-HDAC inhibitor, has also been approved by the FDA in 2014 based on the results of the pivotal phase 2 BELIEF trial [35]. A total of 129 patients with R/R PTCL were treated with 1000 mg/m^2^ belinostat on days 1–5 in 21-day cycles. The ORR was 25.8% including 10.8% CRs. The ORR was 23% in patients with PTCL-NOS, 46% in patients with AITL, and 15% in patients with ALK− ALCL. The median DOR was 13.6 months, and median PFS and OS were 1.6 and 7.9 months, respectively. The most common grade 3 to 4 adverse events were anemia (10.8%), thrombocytopenia (7%), dyspnea (6.2%), and neutropenia (6.2%).

The oral class I/II HDAC inhibitor chidamide has been studied in 83 R/R PTCL patients in China in a phase II study, which included patients with PTCL NOS (34%), ALCL (22%), ENKTL, nasal type (20%), or AITL (13%). Patients received chidamide 30 mg orally twice per week. The ORR was 28% including 14% with CR/CRu. Median PFS and OS were 2.1 and 21.4 months, respectively. AITL patients tended to have higher ORR (50%) and CR/CRu rate (40%), as well as more durable responses to chidamide treatment, with a manageable toxicity profile [36].

#### Hypomethylating agents

5-Azacitidine (5-AZA) has shown efficacy in myeloid neoplasms where response rates appear to correlate with *TET2*, *IDH1/2*, and/or *DNMT3A* mutations which regulate DNA methylation levels [37]. It was hypothesized that hypomethylating agents could be effective in TFH-derived PTCL given the abundance of recurrent *TET2*, *DNMT3A*, and *IDH2* mutations. A retrospective French LYSA cohort study reported clinical outcome of 12 patients with R/R AITL (41% with associated myeloid neoplasms) treated with subcutaneous daily injection of 75 mg/m^2^ 5-AZA for 7 consecutive days every 28-day cycle. The treatment appeared to be promising: ORR was 75% including 50% CR, median PFS was 15 months, and median OS was 21 months. Molecular studies revealed *TET2* mutation in 100% of patients, 33% with *DNMT3A* mutations, 41% with *RHOA* mutations, and one patient with *IDH2*^*R172*^ mutation [38]. Currently, a global phase 3 study is ongoing to prospectively compare the efficacy and safety of oral azacitidine (CC486) to the investigator’s single-agent choice of either romidepsin, bendamustine, or gemcitabine in patients with R/R AITL (NCT03593018). Azacitadine has also been studied in combination with romidepsin in a multicenter phase I trial. Azacitadine was given orally at a MTD of 300 mg on days 1 to 14 with romidpesin 14 mg/m^2^ given IV on days 8, 15, and 22 on a 35-day cycle in 31 patients with R/R lymphomas. This pair was particularly effective in PTCL, where it produced ORR of 73% with 55% CR and was well tolerated [39]. A phase II study with this combination is maturing to confirm the efficacy data in PTCL.

### Targeting tumor microenvironment

#### PD1/PD-L1

PD1 is a type I membrane protein that belongs to the immunoglobulin superfamily. It is a member of the extended CD28/CTLA-4 family of immune checkpoint that guards against autoimmunity by downregulating the immune system and promoting self-tolerance [40]. PD-1 is expressed on the surface of activated T cells, B cells, and macrophages and negatively regulates immune responses broadly. PD1 binds two ligands, PD-L1 and PD-L2. In PTCL, PD-1 expression was detected in atypical T lymphoma cells in the majority of AITL (> 90%), to a lesser extent in PTCL/NOS (30–60%), and rarely in other subtypes [41]. In AITL, both PD1 and CXCL13, a chemokine promoting B cell migration and survival in lymph node, showed a similar expression frequency highlighting follicular helper T cells (TFH) [42], which may share additional TFH markers including CD10, BCL6, and ICOS [43]. In NKTCL, PD-L1 expression in NK/T cell lymphoma cells ranged from 56 to 93% in various studies, while PD-1 level was low [44]. In ATLL, PD-L1 amplification was shown as a strong genetic predictor in both aggressive and indolent ATLL [45]. Anti-PD1 immune checkpoint blockade strategy has generated mixed results in PTCL to date due to the complexity that malignant T cell lymphoma cells can express PD-1. In such instances, immune checkpoint blockade has the potential to accelerate tumor growth in tumor cells with high PD-1 expression, as proposed in murine studies [46]. ATLL patients treated with nivolumab developed rapid disease progression after 1 cycle of nivolumab treatment [47], cautioning against the use of immune checkpoint blockade in ATLL without further understanding the complex mechanism of immune evasion and activation. In other subtypes, responses have been mixed. In patients with R/R T cell lymphoma, single-agent nivolumab produced ORR of 40% [48], while PD1 blockade with pembrolizumab showed high efficacy (100% ORR, 71% CR) in a case series of 7 R/R EBV-associated NK and T cell lymphoma [49]. A phase II multicenter trial of patients with R/R PTCL who were treated with pembrolizumab was halted early after a preplanned interim futility analysis [49]. Nevertheless, several trials are ongoing to further explore the efficacy of PD-1 blockade in PTCL. Pembrolizumab is being added to romidepsin (NCT03278782) and pralatrexate (NCT03598998) in ongoing phase I/II trials in patients with R/R PTCL. Pembrolizumab is also being combined with pralatrexate and the epigenic modifier decitabine in an ongoing phase I trial (NCT03240211). The PD-1 monoclonal antibody durvalumab is being studied alone and in combination with lenalidomide in patients with R/R PTCL and CTCL (NCT03011814) as well as in combination with pralatrexate, romidepsin, and/or azacitdine (NCT03161223). The novel PD-1 monoclonal antibodies avelumab (NCT03046953) and tislelizumab (NCT03493451) are being evaluated in R/R PTCL and R/R mature NK and T cell lymphomas, respectively.

#### Immunomodulatory agents

Lenalidomide is a well-established immunomodulatory agent in B cell NHL and multiple myeloma, which has shown modest single-agent activity in PTCL in phase 2 studies. The EXPECT trial with lenalidomide 25 mg given once daily on days 1–21 of 28-day cycle showed ORR of 22% with 11% CR/Cru in various R/R PTCL subtypes, including ORR 31% with CR/Cru 15% in AITL [50]. Overall, 35% of patients experienced at least 1 AE that lead to study dose interruption or reduction, most commonly neutropenia or thrombocytopenia. Lenalidomide given at 25 mg daily continuously in a phase 2 study of Japanese patients with R/R ATLL showed ORR of 42% with 19% CR/CRu, and median PFS and OS at 3.8 and 20.3 months, respectively. Toxicities were manageable in this population with all serious AEs resolving or resolved by the end-of-study assessment [51].

### Targeting signaling and proliferation pathways

#### ALK

Anaplastic lymphoma kinase (ALK) is a receptor tyrosine kinase known to be oncogenically activated in a subset of ALCL. Crizotinib is an ALK-ROS1-MET small molecule inhibitor which showed a high response rate (ORR 90%) in the Children’s Oncology Group study of 26 ALK+ ALCL patients [33, 52]. In this prospective phase I/II trial, patients were treated with crizotinib at 165 mg/m^2^ or 280 mg/m^2^ orally twice daily on days 1–28 of 28-day cycles. CR was observed in 83% (five of six) of ALCL165 and 80% (16 of 20) of ALCL280, with a median duration of therapy of 2.79 and 0.4 years, respectively. A retrospective study of 11 adult patients with R/R ALK+ lymphoma treated with crizotinib 250 mg twice daily until disease progression achieved 90.9% ORR with OS and PFS at 2 years of 72.7% and 63.7%, respectively [53]. Crizotinib-related toxicities were mild and manageable. These studies highlight the important role the ALK pathway plays in these diseases.

#### PI3K inhibitors

Phosphatidylinositol 3-kinase (PI3K) is a lipid kinase that is involved in intracellular signal transduction, with the PI3K-δ and PI3K-γ isoforms preferentially expressed in leukocytes where they modulate both innate and adaptive immune function by mediating pathways important for survival, proliferation, and differentiation [54, 55]. Specifically, inhibition of PI3K-δ and PI3K-γ isoforms blocks mitogenic and survival within tumor cells, disrupts interaction with the tumor microenvironment, and restores antitumor immune responses. The PI3K δ/γ inhibitor duvelisib was evaluated in a phase I dose-escalation trial in 16 patients with R/R PTCL at a MTD of 75 mg twice daily on a 28-day cycle where it resulted in a 50% ORR with 3 CRs. The median PFS was 8.3 months, with activity demonstrated across a number of subtypes [56], and a phase II trial is underway (NCT03372057). Duvelasib is also being studied in combination with romidepsin and bortezomib in a parallel phase I study in patients with PTCL and CTCL. Duvelasib is given at 75 mg twice daily on days 1–28 on 28-day cycles with either romidepsin 10 mg/m^2^ on days 1, 8, and 15 (arm A) or with bortezomib 1 mg/m^2^ on days 1, 4, 8, and 11 (arm B) [56, 57]. These combinations show activity with ORR and CR rates of 55% and 27% for patients with PTCL treated on arm A, and 36% and 21%, respectively, in patients treated on arm B. The median PFS and OS were 8.8 months and 9.1 months for PTCL patients on arm A and 3.5 months and 9.3 months for patients on arm B. Of note, 65% of patients treated on arm A experienced grade 3 or higher adverse events (AEs) with neutropenia (18%) and elevated transaminases (15%) being the most frequent. Among patients treated on arm B, 45% experienced grade 3 or higher AEs with neutropenia (18%) occurring in more than 10% of patients. A phase IB dose-escalation study is being planned to examine the combination of romidepsin with the PI3K α/δ inhibitor copanlisib (NCT04233697). The PI3K-δ inhibitor YY-20394 is being evaluated in an ongoing phase I trial in patients with R/R PTCL (NCT04108325).

#### mTOR inhibitors

Everolimus is an oral agent that inhibits the mammalian target of rapamycin (mTOR) pathway and has been shown to strongly inhibit malignant T cell proliferation in vitro [58]. Single-agent everolimus given at 10 mg daily continuously has been studied in a phase II trial including 16 patients with R/R PTCL with a variety of subtypes, including 4 PTCL-NOS, 2 ALCL, and 1 each had extranodal NK/T cell, AITL, and precursor TLL lymphoma. Everolimus resulted in an ORR of 44% with a median PFS of 8.5 months and an overall survival of 10.2 months. Among responders, the median DOR was 8.5 months [58]. Dose reduction to 5 mg daily was required in 35% of patients.

#### Aurora kinase inhibition

Aurora A kinase (AAK) is a mitotic oncogenic serine/threonine kinase that plays an essential role in progression through the cell cycle during mitosis and has been shown to be upregulated in aggressive lymphomas [59–61]. Alisertib is an oral AAK inhibitor that was studied in the SWOG1108 phase II trial which included 37 patients with R/R PTCL or transformed mycoses fungoides [62]. Patients received alisertib 50 mg twice daily for 7 days every 21-day cycle, resulting in a 30% ORR with 7% CRs, and median PFS and OS of 3 months and 8 months respectively [64]. Consistent with earlier phase I data, myelosuppression was common (in up to 32% of patients) and was the most common reason for dose reductions. The phase III Lumiere trial compared single-agent alisertib to investigator’s choice of single-agent of pralatrexate, gemcitabine, or romidepsin [63]. Alisertib showed similar results with an ORR of 33% with 16% CR, a median PFS of 2.7 months, and a median OS of 9.9 months. Based on preclinical data suggesting synergism [64], alisertib was studied in combination with romidepsin in a phase I trial of R/R aggressive lymphomas including 4 patients with PTCL. While the primary endpoint of the study was to demonstrate safety and tolerability, this combination resulted in disappointing results in these heavily pretreated PTCL patients, with no CRs or PRs [65].

#### Antifolates

Pralatrexate, a folate analogue metabolic inhibitor that selectively enters cells through reduced folate carrier type 1 (RFC-1), was studied in the pivotal multicenter phase II PROPEL trial [66]. Patients with R/R PTCL were treated with weekly pralatrexate 30 mg/m^2^ infusions for 6 weeks in 7-week cycles. The response rate among the 109 evaluable patients was 29%, including 12 complete responses (11%) and 20 partial responses (18%), with a median DoR of 10.1 months. Median PFS and OS were 3.5 and 14.5 months, respectively. Pralatrexate received FDA’s approval for R/R PTCL in 2009.

## Therapies available and under investigation to target biomarkers in frontline PTCL

CHOP (cyclophosphamide, doxorubicin, vincristine, and prednisone) is the most commonly prescribed initial therapy for PTCL, despite results showing that the majority of PTCL patients have an inferior outcome compared with their B cell counterparts receiving CHOP, with the exception of ALK-positive ALCL. Data from the international T cell project showed that the 5-year OS for PTCL-NOS, AITL, and NKTCLs was 32% compared with 14% for ATLL. ALK-positive ALCL demonstrated 5-year OS of 70%, compared to 49% with ALK-negative ALCL [1]. There is a paucity of prospective randomized trials comparing alternative chemotherapy combinations with CHOP in frontline setting, given disease rarity and heterogeneity. The German High-Grade Non-Hodgkin Lymphoma Study Group, in a retrospective ad hoc analysis of over 300 patients with PTCL, showed that the addition of etoposide to CHOP (CHOEP) improved the EFS of patients younger than 60 who had ALCL and normal LDH levels. Other subgroups of patients did not appear to significantly benefit from this addition [67]. To date, standard-dose CHOP remains the reference regimen in PTCL treatment. Efforts to improve frontline therapy in PTCL have been focused on several strategies: (1) to improve upon CHOP by incorporating novel agent X into CHOP chemotherapy backbone, whereas X denotes therapeutic targeting of surface biomarkers such as CD30 and CD52, or epigenetic modifiers regulating essential pathogenic pathways involving modification of histone acetylation and methylation; (2) to explore novel combination free of conventional chemotherapy; and (3) to experiment with novel agents for consolidation and maintenance following chemotherapy induction.

### Biomarker-driven strategies to improve CHOP-based induction chemotherapy

#### Targeting CD30-positive PTCL with brentuximab vedotin plus chemotherapy

The feasibility of adding BV to CHOP in first-line setting was evaluated in a phase 1 study with BV 1.8 mg/kg administered either sequentially with standard-dose CHOP (BV × 2 cycles, followed by CHOP × 6 cycles) or in combination with CHP (CHOP without vincristine) for 6 cycles in patients with mostly CD30-expressing ALCL. Responders received single-agent brentuximab vedotin for 8–10 additional cycles (for a total of 16 cycles) [68]. The ORR was 85% (CR 62%) with sequential treatment, and ORR was 100% (CR 88%) with combination treatment. Safety profile was manageable. Durable remission at 5 years was noted in 50% of patients without subsequent anti-cancer therapy [69]. Based on this encouraging phase 1 data, a global double-blind, randomized phase 3 study (ECHELON-2, NCT01777152) was initiated in 2013 comparing BV-CHP with standard CHOP in untreated CD30-positive PTCL (targeting 75% ALCL) [70], and randomized 452 patients. BV-CHP was associated with higher CR rate (68% vs 56%, *p* = 0.0066) and superior survival. Median PFS was 48.2 months in the BV-CHP group, significantly improved compared to 20.8 months in the CHOP group. In addition, BV-CHP reduced the risk of death by 34% compared with CHOP. Adverse events, including febrile neutropenia and peripheral neuropathy, were similar between groups. ECHELON-2 is the first randomized phase 3 study establishing the superiority of BV-CHP over CHOP in untreated PTCL, which led to FDA approval in November 2018 of BV-CHP as frontline therapy for CD30-positive PTCL. Although the ECHELON-2 study included non-ALCL cases such as PTCL-NOS, AITL, ATLL, and EATL (~ 30% enrollment, 136 total), their small sample sizes precluded sufficient power analysis.

#### Targeting CD52-positive PTCL

Given the variable expression of CD52 in PTCL subtypes, a prospective multicenter trial evaluated the feasibility and clinical efficacy of the combination of alemtuzumab with CHOP regimen (CHOP-C) as the primary treatment for 24 patients with peripheral T cell lymphoma (PTCL) that included PTCL/NOS, AITL, ALK-negative ALCL, and EATL [71]. Patients received 8 CHOP courses, with alemtuzumab given at 30 mg subcutaneously at day 1 prior to CHOP given at standard 21-day cycle. Complete remission (CR) was achieved in 17 (71%) patients, 2-yr OS was estimated at 53% and 2-yr PFS at 48%. Despite promising CR rate, alemtuzumab-containing therapy was accompanied by substantial immunosuppression and infectious complications. Similar findings were noted in more intensive combinations of alemtuzumab plus either CHOP-14 (HOVON) [72] or DA-EPOCH (etoposide, prednisone, vincristine, cyclophosphamide, and doxorubicin) [73]. Twenty patients were treated on the phase 2 HOVON alemtuzumab-CHOP14 study, which showed ORR of 90%; CR 60%; 2-year OS and EFS at 55% and 27%, respectively; high rates of neutropenic fever (40%); and CMV reactivation (35%), as well as secondary EBV-related lymphoma (15%) [72]. Thirty patients were treated on the phase 1/2 NCI alemtuzumab with DA-EPOCH study. CR was achieved in 17 (57%) patients, and ORR was 83%, with median OS and PFS at 20.2 and 6.6 months, respectively. There were five treatment-related deaths on study due to infectious complications [73]. Phase 3 studies comparing the addition of alemtuzumab to CHOP14 versus CHOP14 alone in younger (ACT-1, NCT00646854) and elderly (ACT-2, NCT00725231) patients could not reach planned sample sizes due to low recruitment, and combined analysis of ACT-1 and ACT-2 studies of 252 patients showed no significant difference in EFS, PFS, and OS, while alemtuzumab-CHOP was associated with more infections.

#### Targeting epigenetic pathways

##### HDAC inhibitors

Feasibility of HDAC inhibitors with CHOP combination has been evaluated with romidepsin, belinostat, and chidamide in the first-line setting. In the phase Ib/II LYSA study of romidepsin-CHOP combination, the RP2D for romidepsin was 12 mg/m^2^ on days 1 and 8 of each cycle for 8 cycles. High CR rate (51%) was achieved with 30-month PFS of 41% and 30-month OS of 70.7% [35], which forms the basis for the ongoing phase 3 LYSARC study (NCT01796002) evaluating survival and response with 6 cycles of Ro-CHOP compared to standard CHOP. The outcome of this phase 3 study is maturing and highly awaited, especially with regard to non-ALCL PTCL subtypes including AITL in the context of ECHELON-2 data which enrolled mostly ALCL patients. The addition of romidepsin to CHOEP is also being evaluated in ongoing phase I/II study in young patients with nodal PTCL prior to proceeding with hematopoetic stem cell transplant (NCT02223208).

The combination of belinostat plus CHOP in frontline setting was explored as well, with phase 3 study of Bel-CHOP planned [74]. The oral HDAC inhibitor chidamide was evaluated in a phase 1b/2 study in combination with CHOP in China [75]. Patients received one dose chidamide as the run-in treatment, followed 4 days later by CHOP (6 cycles, each 21 days) with chidamide on days 1, 4, 8, and 11 at MTD of 30 mg. Patients achieving a CR/Cru following induction treatment received consolidation treatment with chidamide on days 1, 4, 8, and 11 every 21 days for 24 months maximum. Overall, 46.6% achieved CR/Cru with an additional 35.7% of patients achieving a PR, and PFS and OS at 12 months were 54.3% and 100%, respectively. Fifteen patients entered the consolidation phase. The combination was well tolerated with no AEs leading to discontinuation.

##### Hypomethylating agents

In the frontline setting, a phase 2 multi-center trial evaluating the combination of oral azacitidine (CC486) plus CHOP as initial therapy for PTCL is ongoing, which prioritizes enrollment of PTCL patients with T follicular helper (TFH) phenotype and acquired genetic predisposition to chemo-sensitization by hypomethylating agent (NCT03542266). Treatment program consists of priming of oral azacitidine for 7–14 days prior to each cycle of standard CHOP.

#### Targeting tumor microenvironment

Lenalidomide in combination with CHOP-based chemotherapy was evaluated in 2 prospective phase 2 studies; both showed unexpectedly modest CR and PFS rates and high discontinuation rates due to substantial toxicities. The T cell Consortium study combined lenalidomide given 10 mg on days 1–10 with standard-dose CHOEP (cyclophosphamide, doxorubicin, etoposide, vincristine, and prednisone) every 21-day cycle for 6 cycles followed by either upfront ASCT or lenalidomide maintenance (NCT02561273). The phase 2 efficacy analysis showed ITT ORR of 68% with CR of 48% and 1-year PFS and OS at 68% and 89%, respectively. Serious AEs (SAEs) and AEs included 5 treatment-related deaths and significant grade > 3 cytopenias [76]. Lenalidomide (25 mg/day on days 1–14) plus CHOP was studied in AITL elderly patients by the French LYSA group (NCT01553786) [77]. ITT CR in the Len-CHOP study was 43.6%, with a 2-year PFS rate of 42.3% and a 2-year OS of 60.1%. Discontinuation rate was high at 28% due to toxicities and disease progression. Mutational analysis demonstrated *TET2* mutations in 49 cases (77%), *RHOA*^*G17V*^ mutations in 34 patients (53%), *DNMT3A* mutations in 20 (31%) patients, and *IDH2* mutations in 14 (22%).

#### Antifolate combination

The antifolate agent pralatrexate was incorporated into the front-line setting alternating with CEOP (cyclophosphamide, doxorubicin, vincristine, and prednisone) in a phase II T cell Consortium trial [78]. Of 33 patients, 21 had PTCL-not otherwise specified (64%), 8 had AITL (24%), and 4 had ALCL (12%). Fifty-two percent of patients achieved a CR. The 2-year PFS and OS were 39% and 60%, respectively. Overall, this approach did not improve outcomes compared to historical data using CHOP.

### Novel combination free of conventional chemotherapy

Given the efficacy of single-agent immunomodulatory agent lenalidomide and HDAC inhibitor romidepsin in PTCL, the combination of immunomodulation and epigenetic manipulation has the potential synergy to target both tumor microenvironment and tumor cells. Based on a phase I/II trial evaluating the combination of lenalidomide and romidepsin at the MTD of romidepsin 14 mg/m^2^ IV on days 1, 8, and 15 and lenalidomide 25 mg oral on days 1–21 of a 28-day cycle, which demonstrated ORR of 50% with acceptable safety profile in R/R PTCL [32], a multi-center phase 2 study is ongoing to assess the efficacy and safety of the romidepsin and lenalidomide combination in previously untreated PTCL patients who are not chemotherapy candidates (NCT02232516). Treatment is planned for up to 1 year in the absence of disease progression or unacceptable toxicities.

### Biomarker-driven consolidation and maintenance

High-dose chemotherapy and autologous stem cell transplant (HDT-ASCT) for chemotherapy-sensitive disease is a therapeutic strategy for PTCL patients in first remission based on phase 2 studies. Three-year PFS with CHOP-based induction followed by HDT-ASCT was reported in the range of 36–44% in prospective studies [79, 80] and 58% in retrospective series [81, 82], signaling ongoing unmet needs for PFS improvement. Romidepsin maintenance therapy is being evaluated in a multi-center phase 2 study for patients in CR1/PR1 who have completed CHOP-based chemotherapy induction followed by HDT-ASCT with BEAM conditioning (NCT01908777). The primary objective is to assess 2-year PFS. The T cell Consortium frontline study with lenalidomide plus CHOEP has a built-in design of consolidation with either upfront ASCT or lenalidomide maintenance (NCT02561273). Of the responding patients (68%; *n* = 30), 10 proceeded to lenalidomide maintenance. The 1-year estimated PFS and OS are 68% and 89%, respectively, and longer follow-up is needed to assess durability of lenalidomide maintenance treatment [76]. Maintenance strategy without HDT-ASCT was explored in the phase 1 frontline treatment of brentuximab vedotin (BV) plus CHOP combination. Patients received single-agent BV maintenance for 8–10 cycles following CHOP chemotherapy [68]. In the 26 patients (19 ALCL) treated with BV + CHP combination therapy and BV maintenance, ORR was 100% with CR of 92%, and 5-year PFS and OS were 52% and 80%, respectively, demonstrating durable remissions [69]. Ultimately, measurable improvement with consolidation and maintenance therapy will require confirmation from prospectively designed, randomized phase 3 studies.

## Future directions

Emerging novel agents and combinations are in active clinical development which target the epigenome, proliferative signaling pathways, and the tumor microenvironment. Given the frequency of alterations in histone methylation and acetylation genes in PTCL, EZH2, which encodes the histone-lysine N-methyltransferase enzyme, has emerged as a potential therapeutic target. The EZH1/2 inhibitor DS-3201b has demonstrated in vitro anti-tumor activity in lymphoma cell lines and is being investigated in a phase I trial of patients with R/R PTCL (NCT02732275). The IDH2 inhibitor AG-221 is being studied in patients with advanced solid tumors or gliomas or R/R AITL who harbor these mutations (NCT02273739). An ongoing phase II trial is being conducted to explore the efficacy of the JAK1/2 inhibitor ruxolitinib in patients with R/R lymphoma, including PTCL (NCT01431209). Alternative JAK inhibitors are also being investigated including AZD4205 (NCT04105010). The dual SYK/JAK inhibitor cerdulatinib is under evaluation in a phase I/II trial in patients with R/R CLL or NHL, including PTCL (NCT01994382). The BCL-2 inhibitor venetoclax has been approved for use in B cell lymphoma such as CLL and is now being investigated in an ongoing phase II trial in patients with BCL-2-positive TCL, including AITL and TFH origin PTCL (NCT03552692). Tipifarnib, a potent and selective inhibitor of farnesyltransferase, has been shown to modulate CXCL12 signaling pathway and has demonstrated promising preliminary results in patients with R/R AITL and CXCL12+ PTCL with ORR of 45% and a 73% clinical benefit rate [83] in a phase II trial in patients with R/R PTCL (NCT02464228). Engaging the tumor microenvironment remains an attractive approach in PTCL, and various novel strategies are under investigation. ICOS, an important member of the CD28/CTLA-4 family found on TFH cells, is being targeted with the monoclonal antibody MEDI-570 in an ongoing phase I trial in patients with PTCL follicular variant and AITL (NCT02520791). CD38 expression has been demonstrated in up to half of ENKTL [84], and the CD38 monoclonal antibody daratumumab has shown promising ORR of 35.7% in this population [85]. Daratumubab is being combined with gemcitabine, dexamethasone, and cisplatin in patients with CD38-positive PTCL (NCT04251065).

**Table 1:** Licensed agents in PTCL

Agent		Target	Trial/phase	Subtype	***N***	ORR (%), CR (%)	Median DOR (months)	Median PFS(months)	Median OS (months)	AEs
**Frontline**	Brentuximab vedotin + CHP, “BV-CHP”	Anti-CD30 Ab-drug conjugate	ECHELON-2 multinational phase III [70] BV-CHP vs. CHOP	CD30+ PTCL, ALCL 72% (ALK+22%, ALK− 50%), PTCL-NOS 13%, AITL 13%	226	83, 68	Not reported	48.2	Not reached after 3 years follow-up, 34% risk of death reduction	Increased diarrhea, any grade (38% vs 20%) in BV-CHP vs CHOP, other AEs comparable between two cohorts
**Relapsed/refractory**	Brentuximab vedotin	Anti-CD30 Ab-drug conjugate	Multinational phase II [15]	ALCL, ALK+ 28%, ALK− 72%	58	86, 57	12.6	20, 57% at 5 years	Not reached,79% at 5 years	Grade ≥ 3 AEs: neutropenia (21%), thrombocytopenia (14%), peripheral sensory neuropathy (12%)
			Multinational phase II [16]	CD30+ PTCL, PTCL-NOS 63%, AITL 37%	35	41, 24	7.6	2.6	Not reported	Grade ≥ 3 AEs: neutropenia (14%), peripheral sensory neuropathy (9%), and hyperkalemia (9%)
	Romidepsin	HDAC-1 inhibitor	Multinational phase II [30]	PTCL, PTCL-NOS 53%, AITL 21%, ALCL, ALK− 16%	130	25, 15	28	4	11.3	Grade ≥ 3 AEs: thrombocytopenia (24%), neutropenia (20%), infections (all types, 19%)
	Belinostat	Pan-HDAC inhibitor	BELIEF multinational phase II [35]	PTCL, PTCL-NOS 64%, ATIL 18%, ALCL 14% (ALK+ 2%, ALK− 12%), EATL 2%, ENKTL 2%, HSTCL 2%	129	25.8,10.8	13.6	1.6	7.9	Grade ≥ 3 AEs: anemia (10.8%), thrombocytopenia (7%), dyspnea (6.2%), neutropenia (6.2%)
	Chidamide*	HDAC class I/II inhibitor	Chinese phase II [36]	PTCL, PTCL-NOS 35%, ALCL 22% (ALK− 14% ALK+ 4%, ALK unk. 4%), NKTCL 20%, AITL 13%	79	28, 14	9.9	2.1	21.4	Grade ≥ 3 AEs: thrombocytopenia (22%), leukopenia (13%), neutropenia (11%)
	Pralaxtrexate	Antifolate	PROPEL North American phase II [66]	PTCL	109	29, 11	10.5	3.5	14.5	Grade ≥ 3 AEs: thrombocytopenia (32%), mucositis (22%), neutropenia (22%), anemia (18%)
	Mogamulizumab^¥^	Anti-CCR4 mAb	Japanese phase II [27]	CCR4+ R/R ATLL, acute 54%, lymphomatous 23%, chronic 23%	26	50, 31	Not reported	5.2	14.4, 23% at 3 years	All grades: infusion reactions (89%) skin rash (63%, 1 Stevens-Johnson syndrome) Skin rash appears to correlated with response
			Japanese phase II [86]	CCR4+ (≥ 10% by IHC), relapsed PTCL (no refractory pts), PTCL-NOS 55%, AITL 41%, ALCL, ALK− 3%	29	34, 13.5	Not reported	2	14.2	Grade ≥ 3 AEs: lymphopenia (81%), neutropenia (38%), leukopenia (43%) Skin rash (all grades), 51%; grade ≥ 3, 11%

Licensed by FDA unless otherwise noted

*Approved in China

¥Approved in Japan

**Table 2:** Experimental agents and combinations of licensed agents in relapsed/refractory setting

Agent	Target	Trial/phase	Subtype	***N***	ORR (%), CR (%)	Median DOR (months)	PFS (months)	OS (months)	AEs
Alisertib	Aurora A kinase inhibitor	SWOG1108 phase II [62]	R/R PTCL or transformed MF (tMF),PTCL-NOS 35%, AITL 24%, ATLL 11%	37	30, 7	Not reported	3	8	Gr ≥ 3 AEs: neutropenia (32%), anemia (30%), thrombocytopenia (24%), febrile neutropenia (14%), mucositis (11%), rash (5%)
		Lumiere phase III [87],alisertib vs. investigator’s choice	R/R PTCL, PTCL-NOS 45%, AITL 22%, ACLC 6% (ALK− 5% ALK+ 1%), EATL 2%, ENKTL 1%	120	33, 16	5	2.7	9.9	Gr ≥ 3 AEs (alisertib vs. comparator):neutropenia (43% vs 25%), thrombocytopenia (29% vs 27%), anemia (33% vs 11%)
Alemtuzumab	Anti-CD52 mAb	European phase II [20]	R/R PTCL	14	36, 21.4	Not reported	Not reported	Not reported	CMV reactivation in 6 pts Pulmonary aspergillosis in 2 pts EBV-related hemophagocytosis in 2 pts 5 treatment-related deaths
5-Azacitadine	Hypo-methylating agent	Retrospective series [38]	R/R AITL (1 pt previously untreated), (41% with concomitant myeloid neoplasm)	12	75, 50	Not reported	15	21	TET2 mutations detected in all 12 patients
Crizotinib	ALK ROS1 inhibitor	Retrospective series [53]	R/R ALK+ lymphoma, ALCL, ALK+ 82%	11	100, 100	Not reported	63.7% at 2 years	72.7% at 2 years	No Gr ≥ 3 AEs
		Phase I/II Children’s Oncology Group [52]	R/R ALK+ ALCL *Pediatric patients (aged 18 months–22 years old)	26	165 mg/m^2^: 83, 90 280 mg/m^2^: 83, 36	Not reported	Not reported	Not reported	Gr ≥ 3 AEs: 165 mg/m^2^ 22%, 280 mg/m^2^ 70%
Duvelisib	PI3K δ/γ inhibitor	Phase I [56]	R/R PTCL	16	50, 19	Not reported	4.4	Not reported	Gr ≥ 3 AEs: transaminase increases (40% ALT, 17% AST), maculopapular rash (17%), neutropenia (17%)
Everolimus	mTOR inhibitor	Phase II [58]	R/R PTCL + MF,PTCL-NOS 25%, ALCL 12.5%, AITL 6% ENKTL 6%	16	44	8.5	4.1	10.2	Gr ≥ 3 AEs : hematologic toxicity 37.5%
Lenalidomide	Immuno-modulator	EXPECT, phase I/II [50]	R/R PTCL, AITL 48%, PTCL-NOS 37%, ALCL 6%	54	22, 11 AITL: 31, 15	3.6 AITL: 3.5	2.5 AITL: 4.6	Not reported	Gr ≥ 3 AEs experienced by 54% 12 treatment-related deaths: acute respiratory distress syndrome, dyspnea, lung infiltration, neutropenic sepsis, pneumonia and cerebral ischemia (*n* = 1 each)
		ATLL-002 phase II [51]	R/R ATLL, acute 58%, lymphomatous 27%, chronic 15%	26	42, 19	Not reported	3.8	20.3	Gr ≥ 3 AEs: neutropenia (65%), leukopenia (38%), lymphopenia (38%), thrombocytopenia (23%)
Nivolumab	Anti-PDL-1 mAb	Phase Ib [48]	R/R PTCL, AITL 50%, PTCL-NOS 25%, ALCL, ALK− 8%	12	33, 17	3.6	1.9	7.9	Immune-mediated AEs in 34%; 53.5% required treatment for immune-mediated AE
		Phase II [47]	ATLL: 1 acute, 1 lymphomatous, 1 chronic	3	n/a	n/a	n/a	n/a	All 3 patients developed rapid progression of disease after the 1st dose
Pembrolizumab	Anti-PDL-1 mAb	Retrospective series [88]	R/R EBV+ NKTCL, failing L-asparaginase	7	100, 71.4	Not reported	Not reported	Not reported	1 patient (post-alloHSCT) developed grade 2 rash; no other AEs
		Phase II [49]	R/R PTCL	18	33, 27	2.9	3.2	10.6	Trial halted early after preplanned interim futility analysis Grade ≥ 3 rash (17%), grade ≥ 3 pneumonitis (11%)
Romidepsin + henalidomide		Phase I/II [32], phase II ongoing NCT022325-16	R/R TCL, 10 CTCL, 11 PTCL	21	50	Not reported	13.5 weeks*	Not reached	Grade ≥ 3 AEs: neutropenia (48%), thrombocytopenia (38%), anemia (33%), electrolyte abnormalities (K, Phos, glucose, Mg (43%)
Romidepsin + lenalidomide + carfilzomib		Phase Ib/IIa [33]	R/R TCL, PTCL-NOS 47%, AITL 21%	19	50, 31 AITL: 80% CR	9.8 weeks*	9.7 weeks*	Not reached	Grade ≥ 3 AEs in ≥ 10% of patients: neutropenia, thrombocytopenia
Romidepsin + pralatrexate		Phase I/II [34]	R/R lymphomas ,TCL 62%, ATLL 21%, ALCL, ALK− 10%	23	71, 40	4.29	4.4	12.4	Grade 4 AEs: thrombocytopenia (14%), neutropenia (10%), sepsis (7%), fever (3%), and pneumonia (3%)
Romidepsin + azacitadine		Phase I [39]	R/R lymphomas, TCL 35%, AITL 10%, ATLL 6%	31	32, 23 TCL: 73, 55 AITL: 100% CR	2.5; TCL: not reached	Not reached	Not reported	Grade ≥ 3 AEs: thrombocytopenia (27%), neutropenia (42%), lymphopenia (42%), hypotension (12%), hyponatremia (8%)
Duvelisib + romidepsin + bortezomib		Phase I/II [57] Arm A: duvelisib + romidepsinArm B: duvelisib + bortezomib	R/R PTCL + CTCL	Arm A: 22Arm B: 14	Arm A: 55, 27Arm B: 36, 21	Arm A: 8.8 Arm B: 3.5	Arm A: 9.1 (all patients) Arm B: 9.3 (all patients)	Not reported	Arm A: 65% with Gr ≥ 3 AEs: increased ALT/AST (15%), neutropenia (18%), hyponatremia (12%) Arm B: 45% with Gr ≥ 3 AEs: neutropenia (18%), 1 pt with Stevens-Johnson syndrome

**Table 3:** Experimental combinations in the front-line setting

Agent	Trial/phase	Subtype	***N***	ORR (%), CR (%)	Median PFS (months)	Median OS (months)	AEs
Alemtuzumab	Phase II [23]	T-PLL	32	IV route: 91, 81 SubQ route: 33	67% at 12 months	37% at 48 months	IV route: 2 patients with grade 4 hematologic AEs; 2 asymptomatic CMV reactivation; 2 skin reactionsSubQ route: 22% of patients died on treatment
Alemtuzumab + CHOP, “CHOP-C”	GITIL phase II [71]	PTCL, PTCL-NOS 58.3%, AITL 25% ALCL, ALK− 12.5%	24	75, 71	48% at 2 years	53% at 2 years	JC viral encephalitis in 1 pt Invasive aspergillosis in 2 pts PJP in 1 pt Staphylococcus sepsis in 1 pt
CHOP ± alemtuzumab followed by ASCT	ACT-1 phase III [89]	CD 52+ PTCL (no ALCL), PTCL-NOS 58%, AITL 21%	65	77, 52	37% at 3 years	52% at 3 years	Grade 4 leukopenia (73% vs 35% in CHOP arm, *p* = 0.001) Grade ≥ 3 bacterial/fungal infections and other serious AEs similar in both arms *After 2pts developed systemic fungal infections, an amendment tapered ALZ dose from 360 mg (30 mg on days 1 + 2 of each CHOP course) to 120 mg (30 mg on day 1 of CHOP courses 1–4)
Alemtuzumab + CHOP-14	HOVON phase II [72]	CD 52 + PTCL, PTCL-NOS 50%, AITL 30%, subcutaneous panniculitis-like (SPTCL) 15%, EATL 5%	20	90, 60	10	27	Neutropenic fever (40%), CMV reactivation (35%), secondary EBV-related lymphoma (15%)
Alemtuzumab + DA-EPOCH	NCI phase I/II [73]	CD 52+ PTCL, PTC -NOS 35%,ATLL 32%, AITL 13%,CGDTCL 6%,epatosplenic TCL 6%	30	83.3, 57	6.6	20.2	5 treatment-related deaths: 2 sepsis, 1 cardiac arrest, 1 pneumonia, 1 disseminated toxoplasmosis
Romidepsin + CHOP, “Ro-CHOP”, LYSARC	LYSA phase I/II [90]	PTCL, PTCL-NOS 28%, AITL 22% ALCL, ALK− 11%, EATL 6%, follicular PTCL 6%, ATLL 6%	37	69, 51	41% at 30 months	71% at 30 months	Gr ≥ 3 AEs: neutropenia (89%), thrombocytopenia 78%), anemia (43%) QT prolongation < 480 ms (37%); 480–500 ms (5%)
Lenalidomide + CHOP, “len-CHOP”	LYSA phase II [77]	AITL, pts > 59 years old	78	47.4,43.6	42.3% at 2 years	60.1% at 2 years	29% discontinuation rate due to toxicities (15 pts) or POD (8 pts) 4 secondary malignancies 5 treatment-related deaths (4 infections)
Lenalidomide + CHOEP, “len-CHOEP”	T cell consortium phase II [76]	PTCL, PTCL-NOS 57.5%, AITL 30% ALCL, ALK− 12.5%	12	68,48	68% at 1 year	89% at 1 year	25% discontinuation rate due to toxicity (6pts) or POD (4pts) 5 deaths: 1 POD, 2 sepsis, 1 cardiac arrest, 1 secondary malignancy (AML)
Belinostat + CHOP	Bel-CHOP phase I [74]	PTCL, PTCL-NOS 43%, ATIL 39% ALCL, ALK+ 9% ALCL, ALK− 4%	23	89, 72	Not reported	Not reported	Gr ≥ 3 AEs: neutropenia (26%), anemia (22%), lymphopenia (17%)
Pralatrexate alternating with CEOP	T cell Consortium phase II [78]	PTCL,PTCL-NOS 64%, AITL 24% ALC, ALK− 12%	33	70, 52	39% at 2 years	60% at 2 years	Gr ≥ 3 AEs: anemia (27%), thrombocytopenia (12%), febrile neutropenia (18%), mucositis (18%), sepsis (15%), elevated creatinine (12%), elevated and liver transaminases (12%)
Chidamide + CHOP	Phase Ib/II [75]	PTCL ,PTCL-NOS 40%, AITL 26.7% ALCL, ALK+ 13.3% ALCL, ALK− 10%	30	82.1, 46.4	14,54.3% at 12 months	Not reached,100% at 12 months	Gr ≥ 3 AEs: leukopenia (90%), neutropenia (83.3%), lymphopenia (40%), vomiting (13.3%), thrombocytopenia (10%), and febrile neutropenia (10%)

**Table 4:** Novel agents and combinations under investigation in PTCL

Setting	Agent(s)	Study	Phase	***N***	Treatment	**ClinicalTrials.gov**
**Front-line**	Romidepsin + CHOEP	Young patients with untreated nodal PTCL: a phase I–II study	I/II	110	Phase I: romidepsin dose escalation + CHOEP-21, followed by stem cell mobilization and transplantation Phase II: Ro-CHOEP-21 × 3 cycles; if PR or CR, Ro-CHOEP-21 continues for 3 additional cycles followed by stem cell mobilization and transplantation.	NCT02223208
	Azacitadine (CC-486) + CHOP	Previously untreated PTCL	II	20	CC-486 priming is given from D-6 to D0 before cycle 1, and D8-D21 during Cycles 1 to 5.	NCT03542266
	Romidepsin + CHOP vs CHOP	Patients with previously untreated PTCL	III	421	Control Arm: CHOP 21 for 6 cycles Experimental Arm: Ro-CHOP 21 with romidepsin 12 mg/m^2^ given IV on D1 and D8 Q3 weeks for 6 cycles	NCT01796002
	Romidepsin + lenalidomide	Phase II study of romidepsin plus lenalidomide for patients with previously untreated PTCL	II	35	Romidepsin on D1, 8, and 15, and lenalidomide PO QD on D1-21, every 28 days for up to 1 year in the absence of POD or unacceptable toxicity	NCT02232516
**Relapsed/refractory**	Azacitadine vs. investigator’s choice	Randomized phase 3 study for patients with R/R AITL	III	86	Experimental arm: oral azacitadine 300 mg daily × 14 days of 28-day cycles (European patients) Oral azacitadine 200 mg daily × 12 days of 28-day cyclesControl: romidepsin 14 mg/m^2^ on d1, 8, and 15 of 28-day cycle until POD, toxicity, or patient decision, or bendamustine 120 mg/m^2^ on d1 and 2 of a 21-day cycle (during 6 cycles); or gemcitabine 1200 mg/m^2^ on d1, 8, and 15 of a 28-day cycle (during 6 cycles)	NCT03593018
	Pembrolizumab + romidepsin	A phase I/II study of pembrolizumab (MK-3475) in combination with romidepsin in patients with R/R PTCL	I/II	39	Romidepsin is given on D1 and D8. Pembrolizumab is given IV over 30 min on D1. Cycles repeat every 21 days for up to 36 cycles in the absence of POD or unacceptable toxicity.	NCT03278782
	Pembrolizumab + pralatrexate	A phase 1/2 study of pembrolizumab plus pralatrexate for treatment of R/R PTCL	I/II	40	Dose escalation of pralatrexate + pembrolizumab Pralatrexate is given IV over 3–5 min on D1 and 8 and pembrolizumab is given IV over 30 min on D1, repeat every 21 days.	NCT03598998
	Durvalumab ± lenalidomide	A phase 1/2 trial of durvalumab given as a single agent or in combination with lenalidomide in patients with R/R PTCL, including CTCL	I/II	62	Arm 1: patients receive durvalumab IV over 1 h on D1.Arm 2: durvalumab + lenalidomide Patients receive durvalumab IV over 1 h on D1 and lenalidomide PO QD on D1-21.Treatment repeats every 28 days (± 3 days) for up to 13 courses in the absence of POD or unacceptable toxicity for both arms.	NCT03011814
	Durvalumab ± pralatrexate, romidepson, azacitadine	Phase 1/2a study in patients with R/R PTCL	I/II	148	Arm A: durvalumab, 5-azacitadine (AZA)Arm B: durvalumab, pralatrexate, romidepsinArm C: durvalumab, romidepsinArm D: durvalumab, AZA	NCT03161223
	Pembrolizumab ± pralatrexate and decitabine	Novel immuno-epigenetic-based platform for patients with PTCL and CTCL: an international phase Ib study of pembrolizumab combined with decitabine and pralatrexate	Ib	42	Arm A: pembrolizumab + pralatrexate Pembrolizumab 200 mg is given IV D1 1 with pralatrexate 30 mg/m2 IV D1, 8, and 15Arm B: pembrolizumab + pralatrexate + decitabine Pembrolizumab 200 mg is given IV D8 with pralatrexate 20 mg/m2 IV D1, 8, and 15 and decitabine 10 mg/m^2^ from D1 to 5Arm C: pembrolizumab + decitabine Pembrolizumab 200 mg is given IV and decitabine 20 mg/m^2^ from D1 to 5.	NCT03240211
	Avelumab*PD-1 mAb*	A phase 2a trial of avelumab, an anti-PDL1 antibody, in R/R PTCL	II	35	Avelumab is given 10 mg/kg by IV infusion once every 2 weeks. A maximum of 8 cycles, each cycle is 28 days.	NCT03046953
	BGB-A317 (tislelizumab)*PD-1 mAb*	A phase 2, open-label study of BGB-A317 in patients with R/R mature T- and NK-neoplasms	II	90	BGB A317 is given 200 mg IV on D1 of each 21-day cycle.	NCT03493451
	Copanlisib + romidepsin	Copanlisib in combination with romidepsin in patients with R/R mature TCL	IB	30	Dose escalation of copanlisib in combination with romidepsin	NCT04233697
	YY-20394*PI3K-δ inhibitor*	A single-arm, open-label, multi-center, phase I study of YY-20394 in patients with R/R PTCL	I	58	YY-20394 tablets will be given daily for 28 days in 28-day cycles until POD, intolerable toxicity, or the subject discontinues from the study treatment for other reasons.	NCT04108325
	Pralatrexate + romidepsin	Phase I/IIA study of the novel antifolate agent pralatrexate in combination with the histone deacetylase inhibitor romidepsin for the treatment of patients with PTCL	I/II	93	Phase I: dose escalation of pralatrexate and romidepsin. Patients receive both infusions D1 and D15 of each 28-day cycle Phase II: pralatrexate 25 mg/m^2^ and romidepsin 12 mg/m^2^ are given IV once weekly on D1 and 15 on a 28-day cycle	NCT01947140
	DS-3201b*EZH2* inhibitor	A phase 1 multiple ascending dose study of DS-3201b in subjects with lymphomas	I	70	Dose escalation of DS-3201b	NCT02732275
	IDH2 (AG-221)	A phase 1/2, multicenter, open-label, dose-escalation study of AG-221 in subjects with advanced solid tumors, including glioma, and with AITL, that harbor an IDH2 mutation	I/II	21	AG-221 administered orally on every day of 28-day cycles until POD or unacceptable toxicities. Multiple doses.	NCT02273739
	Ruxolitinib*JAK* inhibitor	A phase 2 multicenter, investigator initiated study of oral ruxolitinib phosphate for the treatment of R/R diffuse large B cell and PTCL	II	71	Ruxolitinib is administered orally BID on D1–28 repeat courses Q 28 days in the absence of POD or unacceptable toxicity.	NCT01431209
	AZD4205*JAK* inhibitor	A phase I/II, open-label, multicenter study to investigate the safety, tolerability, pharmacokinetics, and anti-tumor activity of AZD4205 in patients with PTCL	I/II	100	AZD4205 will be administrated orally as capsules in 2 dose cohorts. AZD4205 treatment will be continued until disease progression or intolerable adverse reactions	NCT04105010
	Cerdulatinib*SYK/JAK* inhibitor	A phase 1/2A open-label, multi-dose, multi-center escalation and exploratory study of cerdulatinib (PRT062070) in patients with R/R CLL, SLL, or B cell or T cell NHL	I/II	283	Phase I: Dose escalation or cerdulatiniib staring at 15 mg dailyPhase II: Cerdulatinib administered at 30 mg PO BID for 28-day cycles. Six planned cohorts, cohort 2 also received rituximab IV 375 mg/m^2^	NCT02273739
	Venetoclax*BCL-2* inhibitor	A phase II, open-label, multicenter trial of venetoclax (ABT-199/GDC-0199) as single agent in patients with R/R BCL-2 positive PTCL-NOS, AITL, and other nodal TCL of T-follicular helper origin (TFH)	II	35	Venetoclax (ABT-199) 800 mg is administered orally daily until POD, unacceptable toxicity, withdrawal of consent and/or investigator’s decision	NCT03552692
	Tipifarnib	An open-label phase II study of tipifarnib in subjects with relapsed or refractory peripheral T cell lymphoma	II	30	Tipifarnib 300 mg is given orally twice daily on D1–21 of 28-day treatment cycles	NCT02464228
	MEDI-570 *ICOS mAb*	A phase I trial of MEDI-570 in patients with R/R PTCL follicular variant and AITL	I	46	Anti-ICOS monoclonal antibody MEDI-570 is given IV over 1–4 h on day 1. Treatment repeats every 21 days for up to 12 cycles in the absence of POD or unacceptable toxicity.	NCT02520791
	Daratumumab*CD38 mAb*	A Phase II, open-label, multicenter trial of daratumumab in combination with gemcitabine, dexamethasone and cisplatin (D-GDP) in patients with R/R CD38-positive PTCL-NOS, AITL, and other nodal lymphomas of TFH cell origin	II	35	Induction phase: 4–6 courses (according to response after cycle 4 and to patient compliance) of D-GDP every 21 days as follows: C1: daratumumab 8 mg/kg IV on D2 and on D9 C2-6: daratumumab 16 mg/kg IV on D2 and D9), gemcitabine 1000 mg/sm IV D1 and D8 (gemcitabine on D8 to be skipped in case of grade 3–4 toxicity), cisplatin 75 mg/sm IV D1, dexamethasone 40 mg IV or PO D1-2-3-4-9, G-CSF from D3 to 6, and from D10 to 13 (prolonged if necessary)Maintenance:Starting 28 days after the beginning of C4 or 6 (or in case of toxicity grade > 1, after toxicity is resolved) and up to 24 cycles from start of D-GDP according to the following schedule: daratumumab 16 mg/kg single administration every 2 days	NCT04251065
	ATLCAR.CD30 T cells	Phase II study of the administration of T lymphocytes expressing the CD30 chimeric antigen receptor (CAR) for R/R CD30+ PTCL	II	20	The cellular product consisting of ATLCAR.CD30 cells will be administered via IV injection over 5–10 min through either a peripheral or a central line. The volume of infusion will depend upon the concentration of the cells when frozen and the size of the subject. Administration to eligible subjects will occur within 2–14 days after completing lymphodepleting chemotherapy	NCT04083495
	AMF13	A phase II open-label multicenter study to assess the efficacy and safety of AFM13 in patients with relapsed or refractory CD30-positive peripheral T cell lymphoma or transformed mycosis fungoides (REDIRECT)	II	145	AMF13 is given as weekly IV infusions of 200 mg	NCT04101331
	AMF13	Bispecific antibody AFM13 combined with NK Cells for patients with recurrent or refractory CD30-positive Hodgkin or non-Hodgkin lymphomas	I	30	Patients receive standard of care fludarabine IV over 1 h and standard of care cyclophosphamide IV over 30–60 min on D-4 to -2, AFM13-NK IV over 4 h on D0, and then AFM13 IV over 4 h on D 7, 14, and 21.	NCT04074746
	AUTO4*TRBC1* targeted CAR-T cells	A single-arm, open-label, multi-center, phase I/II study evaluating the safety and clinical activity of AUTO4, a CAR T cell treatment targeting TRBC1, in patients with R/R TRBC1-positive selected T cell non-Hodgkin lymphoma	I/II	55	Following pre-conditioning with chemotherapy (cyclophosphamide and fludarabine) patients are treated with doses from 25 to 225 × 10^6^ RQR8/aTRBC1 CAR T cells. Following phase 2 dose determination, patients will be treated with selected doses of RQR8/aTRBC1 CAR T cells (AUTO4).	NCT03590574

Several innovative techniques are being explored in PTCL. Chimeric antigen receptor T (CAR-T) cells directed against CD30 have been developed and are being explored in a phase II trial (NCT04083495) in patients with R/R CD30+ PTCL. AMF13, a bispecific antibody containing a binding site for CD30 and a second binding site for CD16A, the receptor responsible for NK cell activation, is being evaluated in R/R CTCL (NCT04101331). This molecule is also under clinical investigation in combination with NK cells in patients with R/R CD30+ lymphomas (NCT04074746). The T cell receptor β-chain (TCRB) is a pan-T cell antigen highly expressed on PTCL. CAR-T cells targeting TRBC1 are being studied in patients with TRBC1+ selected R/R PTCL (NCT03590574).

## Conclusion

Peripheral T cell lymphomas are heterogeneous diseases, where relapse and refractory diseases are common. Recent advances in molecular and genomic profiling have provided unprecedented insight into disease pathogenesis driven by distinct cells of origins and molecular pathways. Delivering the most effective treatment tailored to underlying biology and therapeutic targets in the first-line and relapsed settings is poised to make the most enduring impact on patient’s survival outcome.

## Data Availability

Not applicable for this review.

## Abbreviations

AE: Adverse event
AITL: Angioimmunoblastic T cell lymphoma
ALK: Anaplastic lymphoma kinase
ATLL: Adult T cell leukemia/lymphoma
AZA: Azacitadine
BV: Brentuximab-vedotin
CAR-T: Chimeric antigen receptor T cell
CEOP: Cyclophosphamide, etoposide, vincristine, prednisone
CHOEP: Cyclophosphamide, doxorubicin, etoposide, vincristine, and prednisone
CHOP: Cyclophosphamide, doxorubicin, vincristine, prednisone
CMV: Cytomegalovirus
CR: Complete response
CRu: Complete response unconfirmed
CTCL: Cutaneous T cell lymphoma
DOR: Duration of response
EATL: Enteropathy-associated T cell lymphoma
EBV: Epstein-Barr virus
EFS: Event-free survival
ENKTL: Extranodal NK/T cell lymphoma
FTCL: Follicular T cell lymphoma
GEP: Gene expression profile
HDAC: Histone deacetylase
HSTL: Hepatosplenic T cell lymphoma
HTLV-1: Human T cell leukemia virus, type 1
MF: Mycosis fungoides
NGS: Next-generation sequencing
ORR: Overall response rate
OS: Overall survival
PFS: Progression-free survival
PI3K: Phosphoinositide 3-kinase
PJP: *Pneumocystis jiroveci* pneumonia
PR: Partial response
PTCL: Peripheral T cell lymphoma
PTCL-NOS: Peripheral T cell lymphoma, not otherwise specified
R/R: Relapsed/refractory
sAE: Serious adverse event
sALCL: Systemic anaplastic large cell lymphoma
SPTL: Subcutaneous panniculitis-like T cell lymphoma
TFH: T follicular helper
